# Hacking 9-1-1: Infrastructure Vulnerabilities and Attack Vectors

**DOI:** 10.2196/14383

**Published:** 2019-07-09

**Authors:** Mat Goebel, Christian Dameff, Jeffrey Tully

**Affiliations:** 1 Department of Emergency Medicine University of Massachusetts Medical School–Baystate Springfield, MA United States; 2 Department of Emergency Medicine University of California San Diego San Diego, CA United States; 3 Department of Anesthesiology and Pain Medicine UC Davis Medical Center Sacramento, CA United States

**Keywords:** cybersecurity, emergency medical services, emergency medical dispatch, emergency medical service communication systems

## Abstract

9-1-1 call centers are a critical component of prehospital care: they accept emergency calls, dispatch field responders such as emergency medical services, and provide callers with emergency medical instructions before their arrival. The aim of this study was to describe the technical structure of the 9-1-1 call-taking system and to describe its vulnerabilities that could lead to compromised patient care. 9-1-1 calls answered from mobile phones and landlines use a variety of technologies to provide information about caller location and other information. These interconnected technologies create potential cyber vulnerabilities. A variety of attacks could be carried out on 9-1-1 infrastructure to various ends. Attackers could target individuals, groups, or entire municipalities. These attacks could result in anything from a nuisance to increased loss of life in a physical attack to worse overall outcomes owing to delays in care for time-sensitive conditions. Evolving 9-1-1 systems are increasingly connected and dependent on network technology. As implications of cybersecurity vulnerabilities loom large, future research should examine methods of hardening the 9-1-1 system against attack.

## Introduction

9-1-1 call centers are a critical component of prehospital care: they accept emergency calls, dispatch field responders such as emergency medical services (EMS) units, and provide callers with emergency medical instructions before their arrival. The catalyst for creating the 9-1-1 system in the United States came in 1957, when the National Association of Fire Chiefs recommended using a single number to report fires. In 1967, the Federal Communications Commission worked with the American Telephone and Telegraph Company to establish a single emergency number [1]. 9-1-1 was selected because it had never been authorized as an office code, area code, or service code. The first 9-1-1 call was made on February 16, 1968. Later, Congress passed a legislation making 9-1-1 the official emergency number throughout the United States.

Today, emergencies can be reported through 9-1-1 from 96% of the geography of the United States, placing an estimated 240 million calls annually. There are 5783 public safety answering points (PSAPs, colloquially known as 9-1-1 call centers) in 3135 counties throughout the United States [2]. The public relies on the 9-1-1 service to get assistance during their time of need. Similarly, municipalities rely on PSAPs to know what manner of emergencies are occurring where and when, so that appropriate resources can be dispatched—fire services, emergency medical services, and police services. Medical emergencies comprise 64% of emergency calls [3].

Although the original implementation of basic 9-1-1 services relied mostly on telephone technology, enhanced 9-1-1 (E9-1-1), deployed in the 1980s, introduced significant new elements aimed at identifying the caller’s telephone number and location. Around the same time, new tools were developed to assist dispatchers in handling emergency calls: advanced hardware and software to receive and display the caller’s number and location data, computer-aided dispatch (CAD) systems to assign and track field responders, and mapping software to display both caller and responder locations. These tools greatly enhanced emergency response capabilities but required additional computer, network, and server infrastructure beyond the rudimentary elements of the telephone network. As capabilities further expanded, systems that were once safely within the walled garden of the telephone company were increasingly connected to less well-regulated networks such as the public internet. Though undoubtedly valuable from a patient outcome perspective, this also introduced new risks.

According to the Department of Homeland Security, the emergency services sector will likely become more of a target for cyberattacks as systems become more interconnected and dependent on information technology for daily operations [4]. Disruption or unavailability of the 9-1-1 service owing to cyberattacks targeting either data or physical infrastructure has occurred and could delay response to emergencies, potentially threatening life, limb, and property [5]. In this study, we aimed to describe the technical structure of the 9-1-1 call-taking system and describe its vulnerabilities that could lead to compromised patient care.

## Technical Infrastructure

### Landline Emergency Calls

**Figure 1:**
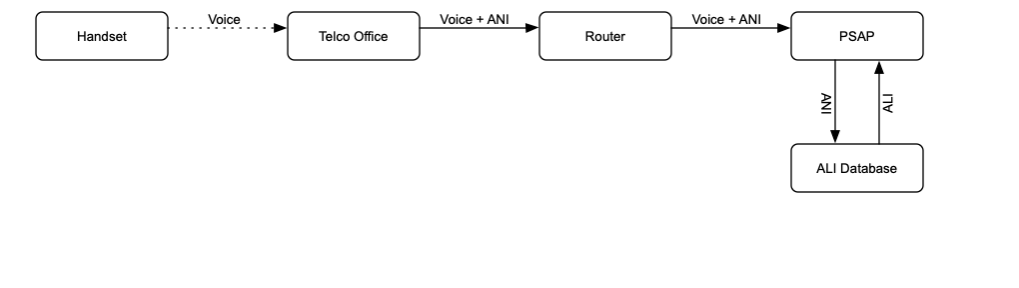
Schema for wired 9-1-1 phone call. ANI: automatic number identification; ALI: automatic location information; PSAP: public safety answering point.

An emergency call placed from a landline is the simplest scenario for emergency call taking (see Figure 1). When the call is placed, the telephone company office (telco) attaches the telephone number to the call. This is called Automatic Number Identification (ANI). The telco sends the call to a router, which forwards the call to the appropriate PSAP (also known as a 9-1-1 call center) based on the ANI. If for some reason the data containing the ANI are corrupted or the call arrives without an ANI, the call is routed to a default PSAP.

The call is answered at the PSAP by a dispatcher, often with the help of a CAD system. One of the dispatcher’s first priorities is to determine the location of the call. The CAD system uses the ANI to search a database, which returns Automatic Location Information (ALI). The ALI record contains the address, phone number, and which services are assigned to the caller’s position.

### Wireless Phase 1 Emergency Calls

**Figure 2:**
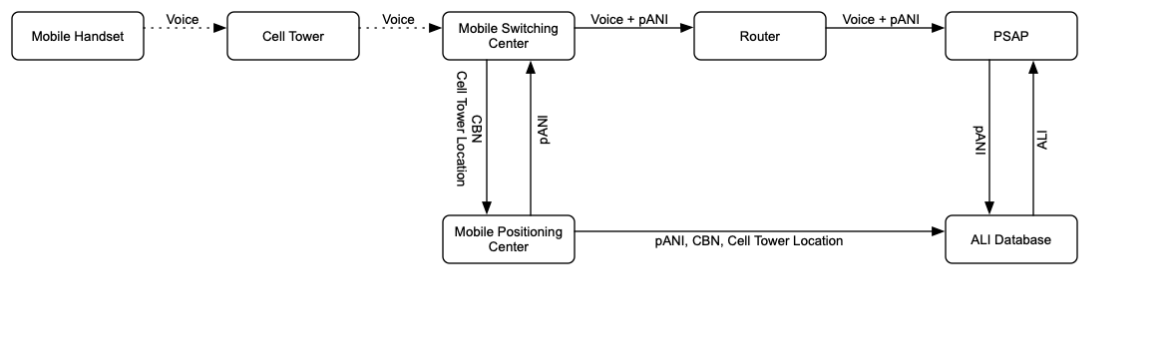
Schema for a phase 1 wireless 9-1-1 call. pANI: pseudo automatic number identification; ALI: automatic location information; PSAP: public safety answering point; CBN: call back number.

A wireless emergency call’s routing varies depending on the E9-1-1 implementation. In a phase 1 implementation, the call is sent from a cell tower to a mobile switching center (see Figure 2). The mobile switching center sends the call and cell tower information to a mobile positioning center. The positioning center returns a pseudo-ANI (pANI), which corresponds to the telephone number of the cell site. The positioning center also creates temporary location information (using the cell tower location) that can be looked up by the PSAP. The switching center attaches the pANI to the call and forwards the call to a router, which selects the appropriate PSAP. The PSAP then looks up the location information, which returns the position approximated by the location of the cell tower.

### Wireless Phase 2 Emergency Calls

**Figure 3:**
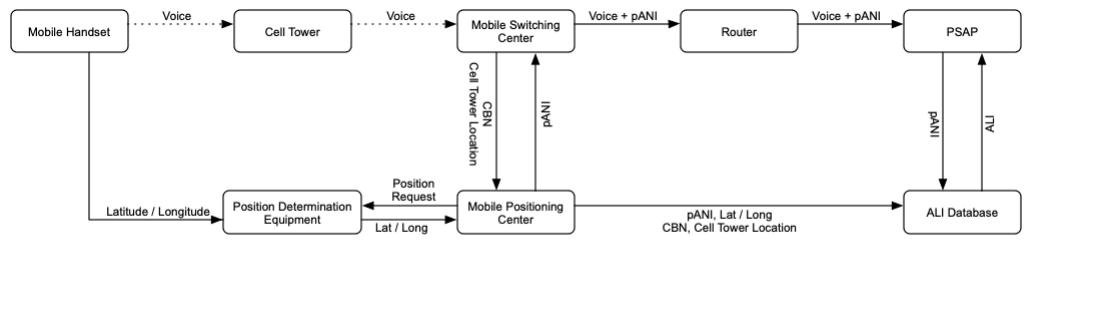
Schema for a phase 2 wireless 9-1-1 call. pANI: pseudo automatic number identification; ALI: automatic location information; PSAP: public safety answering point; Lat: latitude; Long: longitude; CBN: call back number.

In a phase 2 implementation, additional equipment determines the caller’s location more precisely (see Figure 3). The exact equipment and methods vary between cellular providers, but the end relay of position information is the same. When a call is placed, the same chain of events occurs as in phase 1. However, when the mobile positioning center receives the cell tower information, it requests the caller’s location from position determination equipment, which returns latitude and longitude reported by the mobile device. The positioning center returns a pANI as before but enters the actual latitude and longitude of the call in the location database rather than an estimated position based on cell tower information. The switching center sends the call to a router, which selects the appropriate PSAP. When the PSAP queries the location database, the ALI includes the latitude and longitude of the call. In this implementation, a mapping or other geographic information system is commonly integrated with the CAD to automatically plot the call’s location.

### Voice-Over-IP Emergency Calls

**Figure 4:**
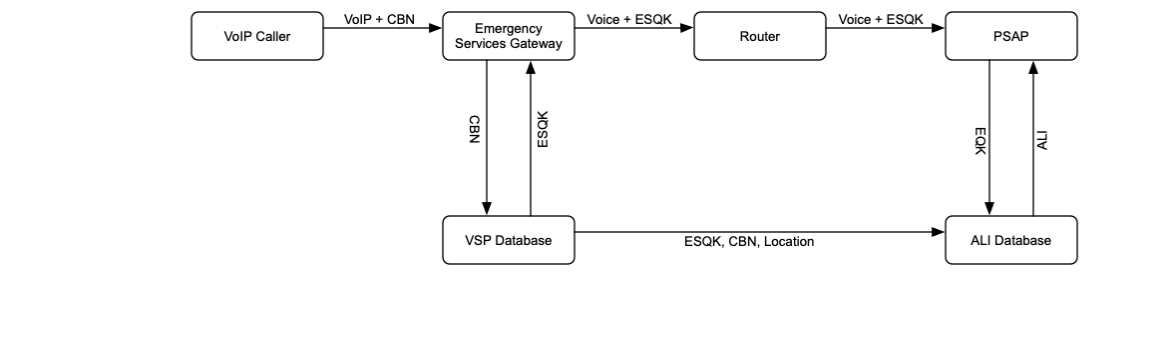
Schema for a VoIP call. VoIP: voice-over-internet-protocol; ESQK: emergency services query key; CBN: call back number; ALI: automated location information; PSAP: public safety answering point; VSP: voice-over-IP service provider.

Voice-over-IP (VoIP), or voice communications delivered by internet-connected networks from providers such as Skype (Skype Technologies, Palo Alto, CA) and Vonage (Vonage Holdings Corp, Holmdel, New Jersey), presents a special problem for 9-1-1 location services (see Figure 4). In this scenario, the VoIP service provider (VSP) maintains a database that associates their subscribers with the appropriate PSAP and location information. These data are usually based on billing information provided by the user. When an emergency call is placed, the call is sent to an Emergency Services Gateway. The gateway searches the VSP database, returning the correct PSAP. The VSP database uses the subscriber’s information to create a temporary location database entry. The call is sent from the gateway to a router, which directs the call to the appropriate PSAP. When the PSAP retrieves location information from the database, it receives the location information provided by the VSP database.

## Vulnerabilities

Information security vulnerabilities fall under 3 main categories: confidentiality, integrity, and availability—often referred to as the CIA triad.

### Breaches of Confidentiality

An attack on confidentiality results in the disclosure of previously private information or communications to one or more parties who are not authorized to receive or view it. Surveillance of the 9-1-1 system could provide attackers with information valuable to planning an incident. Metrics such as call volumes and response times could be used to maximize damage during a physical attack or increase the likelihood of success of criminal acts.

### Breaches of Integrity

An attack breaching integrity violates the correct functioning of a system, producing aberrant results desired by the attacker. An example of this is an attack called swatting, where an individual misdirects law enforcement resources to target another individual, often for personal reasons such as revenge. A December 2017 swatting incident in Wichita, Kansas, led to the shooting of an unarmed man, resulting in his death [6]. Swatting can occur via several methods.

Teletypewriter (TTY) services are intended as communication relays between deaf and hearing persons. A deaf person connects to the relay service using a special terminal with a keyboard or an online service, then requests to an operator who they would like to speak with. The operator connects the call, then verbatim reads messages from the terminal to the caller, and types messages from the caller to the terminal. If an attacker connects to a TTY service and describes an emergency to the operator, the operator will call 9-1-1 and report exactly what the TTY user is typing. Using a TTY service removes some of the attacker’s potentially identifying information (such as their voice). In addition, TTY relay services are required to ensure user confidentiality and are prohibited from keeping records of the contents of any conversation [7]. This method was used to swat Ashton Kutcher and Justin Bieber in 2013 [8].

Other integrity attacks include spoofing apps or services that generate false location or caller identification (ID) information by modifying the ANI or ALI record. This method will not work with a landline because the ANI/ALI is not dynamic. Many spoofing services treat emergency calls differently and will replace the correct ANI data before connecting the call. In addition, when an emergency call is placed from a spoofing service, the service provider ignores any ANI information received from the caller and inserts the correct data. In some rare circumstances, VoIP providers have been found to forward user-provided ANI data without verification or alteration, which could allow spoofing. In addition, this information can seemingly be updated without verification.

If an attacker successfully spoofed ANI information, they could circumvent automatic PSAP routing to target a specific PSAP. The routers that forward emergency calls have a direct dial phone number that is used to transfer calls between PSAPs when a caller is incorrectly routed or if fire and police services are dispatched separately. Although the direct dial numbers for PSAPs’ 9-1-1 network connections and 10-digit emergency lines are kept secret (and are supposed to be nondialable), they can sometimes be discovered during a 9-1-1 call that involves a transfer if the PSAP equipment is not configured to mute the dialing tones used to connect the call. If an attacker utilized a spoofed ANI to call the router at the newly discovered direct dial number, they could place a seemingly authentic call to a specific PSAP. Targeting a specific PSAP could improve an attacker’s chances of getting the desired response to a call, such as a swatting attack.

All location determination mechanisms rely on the PSAP querying the ALI database. If an attacker altered this database, they could change the ALI information for any phone number to their target’s address or change the address associated with their target’s phone number. If an attacker denied access to the ALI database, dispatchers would rely entirely on the information provided by the caller. It is notable that the ALI database represents a single point of failure in every location-determining scheme.

The VSP database that maintains the mapping between the subscriber and location could also be targeted. Modification of the database or a denial of service attack would have the same effect as an attack on the ALI database, but may be secured differently.

Mobile handsets without an active service plan can still be used to place 9-1-1 calls. These nonservice initiated (NSI) devices do not provide a call back number because they do not have a service plan. Instead, they provide a phone number with area code 9-1-1 with the last 7 digits of the device’s electronic serial number or international mobile equipment identity number in place of a phone number. Such a device could be purchased with cash and used to place a call, removing the standard information links between the 9-1-1 call and caller.

Wireless phase 2 calls utilize the location data from the mobile handset itself. It is possible to inject arbitrary coordinates into the handset, either by global positioning system spoofing or modifying the device firmware such that it reports prespecified coordinates. The call will be routed to the appropriate PSAP based on the cell tower location but the ALI record will contain the arbitrary location which is then displayed at the PSAP. This is not foolproof, however, as most cellular providers also use cell tower triangulation as part of the location-determination process.

Attacking the location-determination systems (as described above) or denying service to the entire 9-1-1 infrastructure could delay response to an incident to increase collateral damage. Modifying the VSP or ALI records for an individual could lead to emergency response being directed to the wrong location, delaying emergency services to a targeted individual.

Another integrity attack could target first responders or enhance damage from a physical attack. By misdirecting resources throughout a municipality, an attacker could delay emergency response to a planned physical attack, increasing collateral damage. In addition, an attacker could create a major incident that concentrates emergency responders in a specific location for the purpose of directly attacking responders. These 2 types of attack could also be used in combination. Altering or denying service to the VSP or ALI database could increase the credibility of calls used to initiate such incidents.

### Breaches of Availability

An attack on availability would make a PSAP unavailable to the public. This could be accomplished by a telephone denial of service attack, as occurred during March 2016 in Phoenix. An attacker released a malicious script via a link on Twitter that caused devices to repeatedly call the PSAP without allowing the user to hang up. This produced thousands of false 9-1-1 calls, filling up the phone lines and preventing the public from reporting emergencies [6]. The Federal Bureau of Investigation and Department of Homeland Security have noted an increase in denial of service attacks accomplished by inundating a PSAP’s phone lines with robot dialing [4]. In a December 2018 incident, though not a cyberattack, a failure in the network of internet service provider, CenturyLink, made various PSAPs unreachable in Washington state, Oregon, Minnesota, Massachusetts, Idaho, New Mexico, Missouri, Arizona, and Colorado [9].

## Discussion

### Effects on Patient Care

All 9-1-1–focused attacks to date have been linked to criminal hackers rather than state-sponsored actors [4]. However, as a form of terrorism or even warfare, attacks on the 9-1-1 system could incite panic and loss of life to benefit any enemy of the state. Cyber salvos against the Ukrainian power grid in December 2015, by actors believed to be affiliated with the Russian government, serve as an example of an operation in which an emergency response system may be compromised with failure of underlying infrastructure [10]. Although the direct effects of 9-1-1 attacks on patient care have not been studied, we can infer from other data that delays in emergency care worsens outcomes [11]. Any number of time-sensitive conditions (heart attack, stroke, sepsis, and trauma) could have delayed response times during a 9-1-1 attack, leading to worsened morbidity and mortality.

### Next Generation Attacks

Currently, there is a national effort to upgrade traditional telephone-based 9-1-1 architecture to Next Generation 9-1-1 (NG 9-1-1). This is a collection of technologies that allow PSAPs to receive short message service text messages, images, and even live video streams in addition to existing landline, mobile, and VoIP calls. NG 9-1-1 promises increased capabilities for precisely locating callers and responding to mass casualty incidents, infrastructure disruptions, and natural disasters. However, converting to a system dependent on internet-connected networks raises the possibility of inheriting the same cybersecurity vulnerabilities that plague existing connected infrastructure while continuing to be susceptible to the threat models affecting telephone-based systems that we have already discussed.

Inundating a PSAP with phone calls can make the phone system unavailable. The same attack can be applied to computer systems with the internet or network traffic in a distributed denial of service (DDos) attack. A March 2015 DDos attack on the City of Madison, Wisconsin, targeted municipal websites with the unintended consequence of disrupting 9-1-1 infrastructure. In March 2018, a ransomware attack (malicious software that renders a computer unusable unless an attacker is paid to unlock the system) made the City of Baltimore’s CAD system unavailable to dispatchers for almost 24 hours while systems were restored. Although the PSAP was still reachable, call takers had to gather information by hand that would have normally been displayed automatically, slowing the dispatching process [9].

The National Public Safety Telecommunications Council has outlined the future of EMS communications [5]. Software applications that allow rapid sharing of data between the field and the hospital reduce time to treatment in heart attack and stroke. Sensor networks in vehicles will detect collisions and wearable health devices will detect cardiac arrest to activate 9-1-1 automatically. Prehospital agencies will share telemetry and live video, allowing for physician consultation in the field. Hospitals will have virtual dashboards that give them the status of arriving patients. Although these technologies will be a boon for patient care, adding cyber infrastructure and network interconnection may introduce a variety of vulnerabilities.

Fortunately, centers upgrading to NG 9-1-1 have the potential to mitigate cybersecurity vulnerabilities and related disruptions. Important best practices include frequently patching vulnerable software applications, segmenting networks to protect infrastructure from a direct connection to the internet, and developing incident response plans and teams to react to cybersecurity events that occur [12].

### Future Directions for Readiness

A variety of attacks could be carried out on the 9-1-1 infrastructure to various ends. Attackers could target individuals, groups, or entire municipalities. These attacks could result in anything from a nuisance to increased loss of life in a physical attack to worse overall outcomes owing to delays in care for time-sensitive conditions. Evolving 9-1-1 systems are increasingly connected and dependent on network technology. As implications of cybersecurity vulnerabilities loom large, future research should examine methods of hardening the 9-1-1 system against attack—whether through the technical work of designing and developing increasingly secure software platforms utilizing next generation encryption and authentication methodology to further increase trust of network communications, through implementing regulatory frameworks with incentives for procurement and operational guidance, or through, perhaps most feasible in the short term, raising awareness among individual and regional centers of existing security challenges and corresponding cybersecurity best practices.

## Abbreviations

ALI: Automatic Location Information
ANI: Automatic Number Identification
CAD: computer-aided dispatch
DDos: distributed denial of service
E9-1-1: enhanced 9-1-1
NG 9-1-1: Next Generation 9-1-1
pANI: pseudo-ANI
PSAP: public safety answering point
telco: telephone company office
TTY: teletypewriter
VoIP: Voice-over-IP
VSP: VoIP service provider
